# Conscious Immobility

**Published:** 1964-04

**Authors:** G. de M. Rudolf


### CONSCIOUS IMMOBILITY

BY

G. de M. RUDOLF, M.R.C.P., D.P.M., D.P.H.

A conscious immobility, described first shortly after the end of the last war (Rudolf, 1946a) is a fairly well-known condition in some occupations. It is reasonably frequent amongst printers, and amongst nurses is known as "night nurses' paralysis". This is an inappropriate term as the condition is not exclusively in the night or amongst nurses and it is not a true paralysis.

*Description.*—The sufferer finds suddenly that he, or she, is unable to move, usually, all voluntary muscles. Amongst the Eskimos a somewhat similar condition, known as kayak-angst, kayak-phobia or kayak-dizziness, has been reported (Gussow, 1963). In this state the first symptoms consist of perceptual and cognitive distortions leading to confusion and dizziness. These do not occur in the condition now being discussed. In both states, the onset takes place when the subject is stationary or moving slightly. In the Eskimo, it can begin when he is paddling quietly, and in one male nurse it began as he was slowly turning over the pages of a report book. In the Eskimo, it occurs when the hunter is out in his kayak alone with the sun in his eyes, and the water is calm, mirroring, or slightly wavy. The present condition occurs with the light in any position. The surroundings can be quiet, or noisy as in a printing works. The visually fixed or staring position appears to be necessary for the onset of both conditions. The distortions of kayak-phobia may lead to delusions. These do not occur in the other condition. The subject is fully aware of the environment. In both states, marked fear with the usual accompaniments of sweating, tremor, etc. occur, unless the subject knows that the condition is harmless and will pass away.

In the condition now being discussed, the subject remains normal until he or she wishes to move. This is found to be impossible. In some cases, the inability to move is limited to the eye-muscles only, vision being fixed on a part, or end, of the line of print. In the male nurse already mentioned, the page of the book could not be turned. In most instances, the whole body is immobile.

*Consequences.* Although the condition is harmless, the results of the immobility can be serious. In one instance, a cruiser would have collided with a fully-laden ammunition-ship if an officer had not pushed to one side the navigating officer who had become immobile. Printers have watched paper burning and been unable to move. One printer watched calico being torn. A sergeant-major was unable to fire at the enemy although his rifle was sighted on them and he had been firing shortly before. Trivial results are those of nurses being unable to rise when a sister entered the ward, of a nurse smelling his supper burning and being unable to walk into the kitchen to stop this, and of another who heard a cistern overflowing and could not get up from her chair to pull the chain.

*Differential diagnosis.* The condition is not related to sleep, as is sleep-paralysis, as it occurs when the subject is clearly awake, as on a ship's bridge in daylight. It can be distinguished from voluntary concentration, fear, day-dreaming, sleep, sleep-paralysis, vaso-vagal attacks, hysteria, petit mal, catalepsy, narcolepsy, schizophrenia, post-encephalitic states, coma, concussion and shock (Rudolf, 1947).

*Psychopathology.* In an investigation of eleven subjects (Rudolf, 1946b) who had undergone attacks, slight abnormal signs were found in each, but all were on full naval or military duty overseas and all had been on duty at the times of the attacks. The mild psychopathological states consisted of attacks of depression, chronic anxiety states and over-emotional, hysterical conditions. Nevertheless, many individuals who fall within the wide range of the so-called normal have undergone attacks.

*Knowledge, distribution and frequency.* Knowledge of the existence of the condition varies in different occupations. Of 127 nurses, 41 per cent were aware of it and 21 per cent had passed through attacks; of 21 printers, 24 per cent knew of it and 10 per cent had suffered from it; of 15 Royal Navy executive officers, 33 per cent had heard of it and 13 per cent had had it; of 19 compositors, 5·3 per cent knew of it but none had had attacks; of 60 radar operators none knew of its existence and of 34 doctors only one, a surgeon, knew of the condition. When on a night visit he had seen a nurse unable to move (Rudolf, 1956).

The immobility has occurred in arctic, temperate and tropical climates.

The number of attacks varies in each person. A printer estimated that he had had about 150 in 16 years but a naval officer had had only one in 40 years.

*Termination of attack.* Although, in some cases, numbness may be felt at the end of the attack, the usual ending is for the subject suddenly to find that he can move. Shaking or even touching the subject may abolish the attack but mental confusion has been known to persist for 10 minutes following the disturbance. No confusion occurs if the attack is allowed to pass off spontaneously. Flashing a torch in the sufferer's eyes had no effect in one case and the pupils were found to react to light.

*Causation of attacks.* The cause of the condition is unknown but it may well be that the seat of the trouble is at the myoneural junctions. This is possible as, in some subjects, small jerky movements have occurred immediately before the end of the attacks, and some persons have shaken themselves or shivered just before being able to move.

*Nomenclature.* No appropriate name exists for the condition. Night nurses' paralysis is singularly inappropriate as the condition is not confined to nurses, does not occur in the night only and is not a true paralysis. Tonic immobility and affective hypertonus (Peace, 1954) can be applied to other conditions. A definite name is needed.

### REFERENCES

Gussow, Z. (1963). "Preliminary report of kayak-angst among the Eskimo of West Greenland; a study in sensory deprivation." *Int. J. Soc. Psychiat.*, **9**, 18–26.

Peace, T. (1954). *Lancet*, ii, 1324.

Rudolf, G. de M. (1946a). "'Night-nurse's paralysis': a temporary tonic motor paralysis.' *Bristol med.-chir. J.*, **63**, 132–135.

Rudolf, G. de M. (1946b). "Psychological aspects of a conscious temporary generalized paralysis." *J. ment. Sci.*, **92**, 814–816.

Rudolf, G. de M. (1947). "The differential diagnosis of a conscious temporary generalized motor paralysis." *J. ment. Sci.*, **93**, 369–373.

Rudolf, G. de M. (1956). "A tonic immobility." *Practit.*, **176**, 537–540.

